# 

**Published:** 1845-11

**Authors:** 


TERMS, ONE DOLLAR A YEAR, IN ADVANCE.

THE

### ILLINOIS

### MEDICAL & SURGICAL

### JOURNAL.

EDITED BY
JAMES V. Z. BLANEY, M. D.
**Professor, &c.**

CHICAGO, ILL.
PUBLISHED BY ELLIS & FERGUS,
BOOK AND JOB PRINTERS,
SALOON BUILDINGS, CLARKE STREET,

1845.

VOL. II NO. 8.

